# Psychological Status of Medical Staff in Obstetrics and Gynecology Hospitals during the Omicron Pandemic Outbreak in China

**DOI:** 10.1155/2024/9164605

**Published:** 2024-02-21

**Authors:** Shuting Bao, Bangwu Chen, Ying Hu, Chee Shin Lee, Qi Wu, Menglin Zhou, Mengkai Du, Shuqi Zhu, Biao Xie, Jiuqiong Hu, Zhaoxia Liang

**Affiliations:** ^1^Obstetrical Department, Women's Hospital, School of Medicine, Zhejiang University, Hangzhou, China; ^2^Ninghai Maternal and Child Health Hospital, Ningbo, China

## Abstract

**Background:**

Medical staff in China faced great challenges and psychological and physiological changes of varying degrees during the omicron epidemic outbreak. It is important to recognize the potential impact of these challenges on the mental health of medical staff and to provide appropriate resources and support to mitigate their effects.

**Methods:**

A total of 354 medical staff in two obstetrics and gynecology hospitals of different grades were included in this survey using convenience sampling. The cross-sectional self-report questionnaires survey was conducted using the Basic Characteristics Questionnaire, Generalized Anxiety Disorder (GAD-7), Patient Health Questionnaire (PHQ-9), and Insomnia Severity Index (ISI).

**Results:**

There were 169 (47.7%) participants suffering from anxiety disorder. Working with fever, working in obstetrics, and working with protective clothing were the risk factors for anxiety in medical staff (*p* < 0.05). One hundred and ninety-six (55.4%) participants were depressed. Working with fever and working in obstetrics were the risk factors for depression in medical staff (*p* < 0.05). There were 117 (33.1%) participants suffering from insomnia. Working with fever, high educational level, and severe COVID-19 infection status were the risk factors for insomnia in medical staff (*p* < 0.05). Moreover, medical staff in a provincial hospital were more anxious and depressed than those in a county hospital. At last, there were more participants working with fever in obstetrics (*p* < 0.05).

**Conclusion:**

Anxiety disorder, depression, and insomnia were common among obstetrics and gynecology medical staff during the outbreak of omicron pandemic. During this period, more resources for psychological counselling should be provided to the hospital as well as more reasonable staffing arrangements, and working while having a fever is prohibited, especially in provincial hospital.

## 1. Introduction

Coronavirus disease 2019 (COVID-19) is an emerging infectious disease with the fastest spread, the widest range of infection, and the greatest difficulty in prevention and control in the past century. It is unique and persistent and poses a serious threat to human physical and mental health. The first case of novel coronavirus pneumonia was discovered in December 2019 in Wuhan, China [1]. Over the past three years, China has implemented strict epidemic control measures. On 8 December 2022, the Chinese government issued 10 new epidemic control measures for the novel coronavirus; one of them is “Nucleic acid testing is no longer carried out for the whole population. Except for nursing homes, welfare homes, medical institutions, kindergartners, primary and secondary schools, and other special places, nucleic acid test negative certificates and health codes are not required.” This measure triggers the peak of the omicron epidemic outbreak across the country. During this period, medical personnel in China faced great challenges and psychological and physiological changes of varying degrees. Due to staff shortages, many medical staff go to work despite being sick before their symptoms are completely relieved. Family life, high work intensity, increased work pressure, and even facing the death of patients have led to negative physical and psychological effects on medical staff, such as job burnout, anxiety, depression, and insomnia. Previous surveys and studies in other countries and small outbreaks in China have reported that medical staff experienced varying degrees of anxiety, depression, and insomnia during epidemic outbreaks, which deserves attention [2, 3].

The psychological status of medical staff working in obstetrics and gynecology hospitals is a complex and important issue. The demands of this specialty can be particularly challenging for health professionals, given the emotional intensity of working with women during some of the most vulnerable moments of their lives. The mental health challenges for medical staff in obstetrics and gynecology hospitals can include [4, 5] compassion fatigue and trauma, medical staff may experience emotional exhaustion and a sense of detachment due to constant exposure to traumatic experiences; high-stress situations, medical staff in obstetrics and gynecology hospitals may encounter high-stress situations, such as medical emergencies and complications during childbirth; perinatal loss and grief, medical staff may struggle with their own grief and loss when they are unable to save the life of a patient or her unborn child. It is important to recognize the potential impact of these challenges on the mental health of medical staff and to provide appropriate resources and support to mitigate their effects. However, to date, we have not found any survey and analysis of medical staff working in obstetrics and gynecology hospitals of different grades in China.

The aim of this study was to analyze the psychological status of medical staff in obstetrics and gynecology hospitals in China and its influencing factors during the outbreak of omicron, so as to provide sufficient theoretical and scientific basis for the formulation and implementation of relevant policies and measures to improve the psychological status of medical staff in obstetrics and gynecology hospitals.

## 2. Materials and Methods

### 2.1. Recruitment

The study was conducted through an online survey from January 10 to 31, 2023. A total of 354 respondents were from Women's Hospital, School of Medicine, Zhejiang University (*N* = 166) (a provincial hospital), and Ninghai Maternal and Child Health Hospital (*N* = 188) (a county hospital). This study was approved by the hospital's ethics committee (IRB-20230166-R).

### 2.2. Questionnaire Design

To reduce face-to-face communication and avoid infection, potential respondents were invited electronically, and the response rate was approximately 10%. They completed the questionnaire anonymously through the online survey platform (Surveystar, Changsha Ranxing Science and Technology, Shanghai, China). The questionnaire consists of four parts: basic characteristics, Generalized Anxiety Disorder (GAD-7), Patient Health Questionnaire (PHQ-9), and Insomnia Severity Index (ISI). This questionnaire was used to assess pregnant women's psychological status between December 8 and 31, 2022.

1. Basic characteristics: Gender, age, marital status, level of education, profession, job title, working place, working hours, whether protective clothing was worn, COVID-19 infection status, and whether working with fever were included.
2. Generalized Anxiety Disorder (GAD-7) [6, 7]: The GAD-7 consists of seven items about worry or somatic tension; responses of all GAD-7 items are rated on a 4-point Likert scale as 0 (not at all), 1 (several days), 2 (more than half the days), and 3 (almost every day). Total scores on the GAD-7 therefore range from 0 to 21, with higher scores indicating greater severity of anxiety. For the GAD-7 cut-off points, scores of 5, 10, and 15 generally correspond to the categories of none/normal (0–4), mild anxiety [5–9], moderate anxiety [10–14], and severe anxiety [15–21].
3. Patient Health Questionnaire (PHQ-9) [8, 9]: The PHQ-9 questionnaire consists of nine items, and each item is rated on a 4-point scale from 0 to 3 (0: never; 1: several days; 2: more than half the time; and 3: nearly every day). The total score ranges from 0 to 27, and a score of 10 or greater represents depressive symptoms of at least moderate severity and is the most commonly used cut-off point in screening for major depressive disorder. These scores help to stratify the severity of current depression (minimal, 0–4; mild, 5–9; moderate, 10–14; moderate-to-severe, 15–19; and severe, 20 or more).
4. Insomnia Severity Index (ISI) [10]: The ISI consists of seven items that assess the severity and impact of insomnia, and all items are rated on a five-point Likert scale (0 = no problem; 4 = very severe problem). A total score is obtained after summing up all responses, and the total score ranges from 0 to 28, with 0–7 indicating absence of insomnia, 8–14 indicating subthreshold insomnia, 15–21 indicating moderate insomnia, and 22–28 indicating severe insomnia.

### 2.3. Statistical Analysis

Basic characteristics were described as mean ± SD or number (%), and scores of different questionnaires were also described as mean ± SD or number (%). The F-test was used for parameters with nonnormal distributions. Categorical variables were analyzed using the Chi-square test or Fisher's exact test. Logistic regression was used to estimate odds ratios (ORs) and 95% confidence intervals (CIs) for psychological status in the total participants. *p* < 0.05 was considered statistically significant. Data were analyzed using SPSS software 26.0.

## 3. Results

### 3.1. Basic Characteristics

Basic characteristics of the participants are shown in Table 1. In the provincial hospital, the rate of anxiety disorder, depression, and insomnia were 53.0%, 59.0%, and 35.5%, respectively. While in the county hospital, the rate of anxiety disorder, depression, and insomnia were 43.1%, 52.1%, and 30.9%, respectively. Moreover, there were more participants working with fever in obstetrics than other departments (Table S1). The difference is statistically significant (*p* < 0.05).

### 3.2. The Physiological Status of Medical Staff

The physiological status of medical staff is shown in Table 2. The difference of anxiety disorder and depression between different departments was statistically significant, and obstetrics had the most participants with anxiety disorder and depression (*p* < 0.01, *p* < 0.001, respectively). Participants working with protective clothing were more anxious than those who did not work with protective clothing (*p* < 0.01). Participants who worked with fever were more anxious and depressive than those who did not work with fever (*p* < 0.05, *p* < 0.01, respectively). Table 2 shows that participants who worked with fever were more likely to suffer from insomnia than those who did not work with fever, and the difference is statistically significant (*p* < 0.05). There were no significant differences in the other categories (Table S2, Table S3, and Table S4).

### 3.3. Association between Basic Characteristics and Psychological Status

Tables 3 and 4 show that working with fever, working in obstetrics, and wearing protective clothing were the risk factors for anxiety disorders among medical staff. Working with fever and working in obstetrics were the risk factors for depression. In addition, working with fever, high educational level, and severe COVID-19 infection status were the risk factors for insomnia among medical staff. The difference is statistically significant (*p* < 0.05).

### 3.4. The Comparison of Two Hospitals of Different Grades

As shown in Table 5, medical staff working at Women's Hospital, School of Medicine, Zhejiang University, were more anxious and depressed than those who working at Ninghai Maternal and Child Health Hospital (*p* < 0.05). The working hours of the former were significantly longer than those of the latter (*p* < 0.01).

## 4. Discussion

Psychological distress and sleep problems are important public health issues when epidemics and disasters occur, and these effects can be long lasting. So, we used the form of questionnaire (which includes basic characteristics, GAD-7, PHQ-9, and ISI) to assess the psychological status of medical staff. The current study finds that obstetrics medical staff were more likely to be anxious and depressed and more of them came to work with fever; medical staff who have higher levels of education and who have a severe COVID-19 infection were also more likely to have insomnia; and those who wear protective clothing were more likely to have anxiety disorder. Besides, medical staff of a provincial hospital were more anxious and depressed than those in a county hospital. Moreover, some factors, such as gender, marital status, age, profession, and professional title, had no significant effect.

The current results are in line with prior reports on how COVID-19 has affected medical staff in general hospitals' mental health. About 70% of participants in the other sizable study [2], conducted in China from January 29 to February 3, 2020, reported experiencing distress, with 50% reporting depressed and 34% citing insomnia. Participants from outside Hubei province had a lower likelihood of exhibiting symptoms of distress than those from Wuhan, according to a multivariable logistic regression study. In December 2022, the virus spread throughout all of China at a rate comparable to Wuhan at the time.

There was no discernible variation in psychological status amongst medical staff members of different genders, contrary to earlier reports [11]. This could be for several reasons, including the fact that all medical staff must deal with the same level of responsibility and workload because of the nature and responsibilities of working in obstetrics and gynecology hospitals. In dealing with patients' ailments and pain, medical workers must carry a same emotional load regardless of gender. Male and female medical professionals at obstetrics and gynecology facilities have similar professional demands and challenges, such as long working hours, hectic schedules, ongoing technology advancements, and frequently shifting medical policies. Obstetrics and gynecology hospitals are typically highly specialized medical institutions, and medical staff are required to have the same professional knowledge and skills. This may lead to a narrowing of gender differences in medical expertise. In summary, even though male and female medical staff in obstetrics and gynecology facilities may have distinct working and personal experiences, their job obligations and work demands are comparable; therefore, their levels of anxiety and depression may be fairly balanced.

In this study, working with fever made medical staff more anxious and depressed and sleep deprived. It was hypothesized that having fever and reporting to work could be harmful to the mental health of medical staff. Increased burnout, job stress, and emotional exhaustion are a few of the potential causes. Healthcare organizations should put their employees' health and wellbeing first by advising unwell personnel to stay at home and provide enough assistance and resources to stop burnout and other harmful effects.

The medical professionals in the obstetrics field reported greater incidence of anxiety and depression. Also, a disproportionately greater percentage of medical staff in obstetrics arrive at work with a fever. During the pandemic outbreak, people avoided leaving their homes until absolutely necessary, postponed elective surgeries, and did not seek nonemergency medical attention. During the outbreak, hospitals stopped elective procedures, except in the obstetrics department. Outpatient visits were less than usual. As a result, there were much fewer patients than normal in the gynecological and outpatient clinics. Due to the fact that childbearing is a basic human requirement and pregnancy and childbirth are unavoidable natural processes, the number of obstetric patients will not diminish during the pandemic. The majority of women still require medical services, such as preconception check-ups, pregnancy check-ups and childbirth, even during the epidemic. Also, while the majority of women opt to take antipyretic medications on their own after contracting COVID-19, pregnant women may decide to seek medical attention out of concern for the wellbeing of the fetus. As a result, many women may require more obstetric care to maintain their health and the health of their unborn children. Due to a lack of staff, the obstetric medical staff must report to work when sick. This phenomenon demonstrates the need to fairly adjust each department's employment in light of the epidemic condition.

Although the relationship is complicated and not fully understood, the current finding revealed that there may be a positive association between educational attainment and depression. On the one hand, those with more education typically earn more money, have better employment possibilities, and have access to more tools that can help them deal with stress and other issues in life. Better mental health outcomes and lower incidence of depression may result from this [12, 13]. Higher levels of education, however, can also be accompanied with a variety of pressures and difficulties, including heightened academic pressure, financial strain, and competition for employment possibilities. Increased rates of depression can be brought on by these pressures, particularly in those who may already be predisposed to mental health issues. Overall, the relationship is complex and probably influenced by a number of factors, including personal characteristics, socioeconomic status, and life experiences [14]. There is some evidence to support a positive association between educational attainment and depression.

The degree of education may be positively correlated with insomnia. Also, one explanation for the correlation could be that those with higher levels of education work harder or longer hours, which results in higher levels of stress and trouble unwinding at night. More educated people might also be more prone to partake in activities that interfere with sleep, such as utilizing electronics right before bed. The study demonstrates a negative correlation between utilizing electronic devices before bed and the quality of your sleep [15–17]. More educated medical professionals also have more job obligations. A considerable drop in the number of employees working on the job was caused by the large number of medical professionals who, particularly during the pandemic, took sick leave at home owing to infection. Worries over the general state of the job caused leaders to have insomnia.

Evidently, insomnia is correlated to the severity of COVID-19 infection. It makes sense that these people might experience worse respiratory or fever symptoms, as well as a lengthier illness course, which would have an impact on their quality of sleep. Moreover, according to several studies [18–21], sleep disturbance was the most common neuropsychiatric symptom, and more sleeplessness was linked to a more serious COVID-19 infection.

Previous studies [22–25] suggested that medical staff had anxiety, depression, and even posttraumatic stress disorder (PTSD) after the epidemic outbreak, and few participants had received psychological interventions and fewer participants had received individual interventions. This shows that current psychological interventions were not sufficient, and target psychological interventions should be conducted in time.

In the current study, we compared the mental health of medical staff between two hospitals of different grades, which is the highlight of this article. According to data analysis, medical staff working at Women's Hospital, School of Medicine, Zhejiang University, had higher GAD-7 and PHQ-9 scores than those working at Ninghai Maternal and Child Health Hospital. We could take into account elements like differing working hours, variations in staffing numbers, resources, or patient demographics that would be responsible for these observed discrepancies. Medical staff in provincial hospitals face greater job demands, occupational risk, and social pressure than county hospitals, which could contribute to higher levels of anxiety and depression. A survey found that there is a general shortage of medical staff in tertiary hospitals [26]. In particular, in high-stress work environments, such as tertiary hospitals, it is crucial for healthcare companies to offer support and resources to promote the mental health and wellbeing of their staff.

As far as we are aware, this study is the first of its kind to concentrate on the mental health of medical personnel working in obstetrics and gynecology hospitals during the most recent national COVID-19 pandemic. Also, it is the first-time the hospitals for obstetrics and gynecology of various levels have been compared.

The following limitations must be noted. First, given the study's cross-sectional design, we can only assert association rather than causality, and we hope to be able to follow up the psychological status of medical staff, which is also our future research direction. Second, we have control for some potential confounding factors, such as age and educational level; however, it is really difficult to control demographic characteristics, personality traits, coping styles, social support, and other sources of stress, which may influence the psychological status of the medical staff. Also, because only 354 participants were sampled, there may not be much generalizability. The participants may have been the most affected or those most eager to assist because the responses were voluntary. There is the possibility of a representation bias. This is also a general limitation of questionnaire survey.

## 5. Conclusion

In summary, the psychological status of medical staff working in obstetrics and gynecology hospitals is a critical issue that requires attention and support from healthcare organizations and policymakers. Anxiety disorder, depression, and insomnia are common among medical staff during the outbreak of omicron pandemic in China. More resources for psychological counselling should be provided to the hospital during this time, as well as more reasonable staffing arrangements and a prohibition on working when feverish, especially in Grade A tertiary care facilities.

## Figures and Tables

**Table 1:** Basic characteristics.

Category	Mean ± SD/*N* (%)	
Hospitals^a^	Group 1	Group 2
Gender		
Male	21 (12.7%)	36 (19.1%)
Female	145 (87.3%)	152 (80.9%)
Marital status		
Single	71 (42.8%)	22 (11.7%)
Married	95 (57.2%)	166 (88.3%)
Age		
18–30	83 (50.0%)	30 (16.0%)
31–40	55 (33.1%)	80 (42.5%)
41–60	28 (16.9%)	78 (41.5%)
Level of education		
Below undergraduate	1 (0.6%)	40 (21.3%)
Undergraduate	78 (47.0%)	147 (78.2%)
Master	67 (40.4%)	1 (0.5%)
Doctor or above	20 (12.0%)	0 (0.0%)
Profession		
Medical	104 (62.7%)	139 (73.9%)
Nurse	62 (37.3%)	49 (26.1%)
Job title^b^		
Junior	75 (45.2%)	75 (39.9%)
Intermediate	75 (45.2%)	80 (42.6%)
Senior	16 (9.6%)	33 (17.5%)
Department		
Obstetrics	78 (47.0%)	23 (12.2%)
Gynecology	46 (27.7%)	10 (5.3%)
Outpatient	14 (8.4%)	48 (25.5%)
Others^c^	28 (16.9%)	107 (57.0%)
Wearing protective clothing^d^		
Yes	26 (15.7%)	33 (17.6%)
No	140 (84.3%)	155 (82.4%)
COVID-19 infection status		
Not infected	5 (3.0%)	20 (10.6%)
Asymptomatic infection	1 (0.6%)	3 (1.6%)
Mild type	139 (83.7%)	136 (72.3%)
Common type or more serious	21 (12.7%)	29 (15.3%)
Working with fever		
Yes	95 (57.2%)	86 (45.7%)
No	71 (42.8%)	102 (54.3%)
GAD-7		
GAD-7 < 5	78 (47.0%)	107 (56.9%)
GAD-7 ≥ 5	88 (53.0%)	81 (43.1%)
PHQ-9		
PHQ-9 < 5	68 (41.0%)	90 (47.9%)
PHQ-9 ≥ 5	98 (59.0%)	98 (52.1%)
ISI		
ISI < 8	107 (64.5%)	130 (69.1%)
ISI ≥ 8	59 (35.5%)	58 (30.9%)

^a^Group 1: Women's Hospital, School of Medicine, Zhejiang University; Group 2: Ninghai Maternal and Child Health Hospital. ^b^Job title: junior: responsible for basic medical activities; intermediate: have clinical experience and skills and can perform most medical activities independently; senior: have strong practical experience and skills to perform complex medical activities independently. ^c^Other department: administrative departments, logistics departments, and medical technology auxiliary departments. ^d^Situations require wearing protective clothing: transporting persons of cases and asymptomatic infected persons; environmental cleaning and disinfection personnel; specimen collection personnel; laboratory staff; the staff who conducted epidemiological investigation of suspected, confirmed cases and asymptomatic infection cases; isolation of staff in wards and medical observation places.

**Table 2:** The condition of psychological status.

Category	GAD-7 < 5	GAD-7 ≥ 5	*p*	PHQ-9 < 5	PHQ-9 ≥ 5	*p*	ISI < 8	ISI ≥ 8	*p*
Department			0.001^*∗∗*^			<0.001^*∗∗*^			0.058
Obstetrics	37 (36.6%)	64 (63.4%)		31 (30.7%)	70 (69.3%)		58 (24.5%)	43 (36.8%)	
Gynecology	29 (51.8%)	27 (48.2%)		23 (41.1%)	33 (58.9%)		39 (16.5%)	17 (14.5%)	
Outpatient	42 (67.7%)	20 (32.3%)		40 (64.5%)	22 (35.5%)		48 (20.2%)	14 (12.0%)	
Others	77 (57.0%)	58 (43.0%)		64 (47.4%)	71 (52.6%)		92 (38.8%)	43 (36.7%)	
Wearing protective clothing			0.003^*∗∗*^			0.252			1.000
Yes	20 (33.9%)	39 (66.1%)		22 (13.9%)	37 (18.9%)		40 (16.9%)	19 (16.2%)	
No	165 (55.9%)	130 (44.1%)		136 (86.1%)	159 (81.1%)		197 (83.1%)	98 (83.8%)	
Working with fever			0.026^*∗*^			0.001^*∗∗*^			0.024^*∗*^
Yes	84 (45.4%)	97 (57.4%)		65 (35.9%)	116 (64.1%)		111 (61.3%)	70 (38.7%)	
No	101 (54.6%)	72 (42.6%)		93 (53.8%)	80 (46.2%)		126 (72.8%)	47 (27.2%)	

Categorical variables were analyzed using the Chi-square test or Fisher's exact test. Data are *N* (%). ^*∗*^*p* < 0.05; ^*∗∗*^*p* < 0.01.

**Table 3:** Association between basic characteristics and psychological status.

Category	GAD-7, anxiety		PHQ-9, depression		ISI ≥ 8, insomnia	
	OR (95% CI)	*p*	OR (95% CI)	*p*	OR (95% CI)	*p*
Working with fever						
Yes	1.620 (1.064, 2.466)	0.025^*∗*^	2.075 (1.355, 3.177)	0.001^*∗∗*^	1.691 (1.079, 2.649)	0.022^*∗*^
No	Reference	NA	Reference	NA	Reference	NA
Gender						
Male	Reference	NA	Reference	NA	Reference	NA
Female	0.936 (0.531, 1.650)	0.820	0.885 (0.499, 1.570)	0.675	0.897 (0.495, 1.628)	0.721
Marital status						
Married	Reference	NA	Reference	NA	Reference	NA
Unmarried	0.764 (0.476, 1.228)	0.226	0.720 (0.444, 1.166)	0.182	0.983 (0.595, 1.624)	0.946
Age						
18–30	Reference	NA	Reference	NA	Reference	NA
31–40	0.934 (0.567, 1.540)	0.790	1.190 (0.718, 1.975)	0.500	1.142 (0.672, 1.943)	0.623
41–60	0.673 (0.394, 1.148)	0.146	0.764 (0.449, 1.301)	0.222	1.010 (0.572, 1.783)	0.973
Level of education						
Below undergraduate	Reference	NA	Reference	NA	Reference	NA
Undergraduate	1.572 (0.791, 3.125)	0.197	1.513 (0.774, 2.958)	0.226	3.218 (1.298, 7.980)	0.012^*∗*^
Master	1.950 (0.881, 4.314)	0.099	2.198 (0.998, 4.841)	0.051	2.981 (1.096, 8.114)	0.032^*∗*^
Doctor or above	2.119 (0.715, 6.277)	0.176	2.373 (0.785, 7.177)	0.126	3.889 (1.120, 13.507)	0.033^*∗*^
Profession	1.372 (0.874, 2.152)	0.169	1.086 (0.690, 1.707)	0.722	0.904 (0.559, 1.462)	0.681
Job title						
Junior	Reference	NA	Reference	NA	Reference	NA
Intermediate	1.099 (0.701, 1.723)	0.679	0.980 (0.624, 1.539)	0.931	1.538 (0.949, 2.491)	0.081
Senior	1.011 (0.530, 1.929)	0.974	1.076 (0.561, 2.064)	0.825	1.366 (0.687, 2.718)	0.374
Department						
Obstetrics	2.296 (1.353, 3.898)	0.002^*∗∗*^	2.035 (1.185, 3.497)	0.010^*∗*^	1.586 (0.929, 2.709)	0.091
Gynecology	1.236 (0.661, 2.310)	0.506	1.293 (0.688, 2.430)	0.424	0.933 (0.475, 1.832)	0.839
Outpatient	0.632 (0.336, 1.190)	0.155	0.496 (0.267, 0.922)	0.027^*∗*^	0.624 (0.311, 1.253)	0.185
Others	Reference	NA	Reference	NA	Reference	NA
Wearing protective clothing						
Yes	2.475 (1.377, 4.447)	0.002^*∗∗*^	1.439 (0.809, 2.557)	0.215	0.955 (0.525, 1.735)	0.880
No	Reference	NA	Reference	NA	Reference	NA
COVID-19 infection status						
Not infected	Reference	NA	Reference	NA	Reference	NA
Asymptomatic infection	0.262 (0.024, 2.878)	0.273	1.5 (0.181, 12.459)	0.707	0.000	0.999
Mild type	0.655 (0.287, 1.493)	0.314	1.909 (0.828, 4.399)	0.129	2.639 (0.880, 7.915)	0.083
Common type or more serious	1.085 (0.412, 2.859)	0.869	2.250 (0.844, 5.995)	0.105	3.802 (1.136, 12.720)	0.030^*∗*^

Logistic regression was used to estimate odds ratio (OR) and 95% confidence interval (CI). NA: not applicable. ^*∗*^*p* < 0.05; ^*∗∗*^*p* < 0.01.

**Table 4:** Risk factors for physiological status identified by multivariable logistic regression analysis.

Category	GAD-7, anxiety		PHQ-9, depression		ISI ≥ 8, insomnia	
	Adjusted OR (95% CI)^a^	*p*	Adjusted OR (95% CI)^b^	*p*	Adjusted OR (95% CI)^c^	*p*
Working with fever						
Yes	1.432 (0.923, 2.221)	0.109	1.907 (1.229, 2.959)	0.004^*∗∗*^	1.532 (0.961, 2.442)	0.073
No	Reference	NA	Reference	NA	Reference	NA
Level of education						
Below undergraduate					Reference	NA
Undergraduate					3.250 (1.290, 8.187)	0.012^*∗*^
Master					2.784 (1.004, 7.714)	0.049^*∗*^
Doctor or above					3.395 (0.960, 12.005)	0.058
Department						
Obstetrics	2.027 (1.177, 3.489)	0.011^*∗*^	1.819 (1.048, 3.158)	0.034^*∗*^		
Gynecology	1.222 (0.647, 2.308)	0.536	1.220 (0.644, 2.313)	0.542		
Outpatient	0.558 (0.291, 1.072)	0.080	0.482 (0.257, 0.904)	0.023^*∗*^		
Others	Reference	NA	Reference	NA		
Wearing protective clothing						
Yes	2.306 (1.266, 4.202)	0.006^*∗∗*^				
No	Reference	NA				
COVID-19 infection status						
Not infected					Reference	NA
Asymptomatic infection					0.000	0.999
Mild type					2.445 (0.789, 7.578)	0.121
Common type or more serious					3.628 (1.049, 12.547)	0.042^*∗*^

Logistic regression was used to estimate odds ratio (OR) and 95% confidence interval (CI). ^a^Adjusted for working with fever, department, and protecting wearing; ^b^adjusted for working with fever and department; ^c^adjusted for working with fever, educational level, and COVID-19 infection status; NA: not applicable; ^*∗*^*p* < 0.05; ^*∗∗*^*p* < 0.01.

**Table 5:** The comparison of two hospitals of different grades.

	Group 1	Group 2	*p*
GAD-7	5.37 ± 4.42	3.97 ± 4.10	0.002^*∗∗*^
PHQ-9	6.54 ± 5.15	5.45 ± 4.69	0.038^*∗*^
ISI	6.13 ± 4.75	5.92 ± 5.09	0.687
Working hours per week	47.44 ± 10.23	42.42 ± 13.52	0.000^*∗∗*^

The *F*-test was used for parameters with nonnormal distributions. Data are reported as mean ± SD. Group 1: Women's Hospital, School of Medicine, Zhejiang University; Group 2: Ninghai Maternal and Child Health Hospital. ^*∗*^*p* < 0.05; ^*∗∗*^*p* < 0.01.

## Data Availability

All data generated or analyzed during this study are included within this article and its supplementary information files.

## Supplementary Materials

Supplementary Materials Supplementary tables: some supplementary tables about the condition of psychological status.
