# Supplementary material for: Psychological Status of Medical Staff in Obstetrics and Gynecology Hospitals during the Omicron Pandemic Outbreak in China
Source: Can J Infect Dis Med Microbiol. 2024 Feb 21;2024:9164605. doi: 10.1155/2024/9164605 (PMC10901569; doi:10.1155/2024/9164605)
Supplement: Supplementary Materials — Supplementary tables: some supplementary tables about the condition of psychological status. [file 9164605.f1.docx]

**Supplement Table**

Table S1 The condition of working with fever

| Department | Working with fever | | p |
| --- | --- | --- | --- |
|  | No | Yes |  |
| Obstetrics | 37(36.6%) | 64(63.4%) | 0.018* |
| Gynecology | 26(46.4%) | 30(53.6%) |  |
| Outpatient | 34(54.8%) | 28(45.2%) |  |
| Other | 76(56.3%) | 59(43.7%) |  |

Data were N (%).*P<0.05

Table S2 The condition of anxiety

| Category | | GAD -7 <5 | GAD -7≥5 | P |
| --- | --- | --- | --- | --- |
| Gender |  |  |  | 0.885 |
|  | Male | 29(15.7%) | 28(16.6%) |  |
|  | Female | 156(84.3%) | 141(83.4%) |  |
| Marital status |  |  |  | 0.279 |
|  | Single | 44(23.8%) | 49(29%) |  |
|  | Married | 141(76.2%) | 120(71%) |  |
| Age |  |  |  | 0.297 |
|  | 18-30 | 55(29.7%) | 58(34.3%) |  |
|  | 31-40 | 68(36.8%) | 67(39.6%) |  |
|  | 41-60 | 62(33.5%) | 44(26.1%) |  |
| Level of Education |  |  |  | 0.361 |
|  | Below undergraduate | 26(14.1%) | 15(8.9%) |  |
|  | Undergraduate | 118(63.8%) | 107(63.3%) |  |
|  | Master | 32(%17.3) | 36(21.3%) |  |
|  | Doctor or above | 9(4.9%) | 11(6.5%) |  |
| Profession |  |  |  | 0.171 |
|  | Medical | 133(71.9%) | 110(65.1%) |  |
|  | Nurse | 52(28.1%) | 59(34.9%) |  |
| Job title |  |  |  | 0.911 |
|  | Junior | 80(43.2%) | 70(41.4%) |  |
|  | Intermediate | 79(42.7%) | 76(45%) |  |
|  | Senior | 26(14.1%) | 23(13.6%) |  |
| Department |  |  |  | 0.001** |
|  | Obstetrics | 37(36.6%) | 64(63.4%) |  |
|  | Gynecology | 29(51.8%) | 27(48.2%) |  |
|  | Outpatient | 42(67.7%) | 20(32.3%) |  |
|  | Other | 77(57.0%) | 58(43.0%) |  |
| Wearing protective clothing |  |  |  | 0.003** |
|  | Yes | 20(33.9%) | 39(66.1%) |  |
|  | No | 165(55.9%) | 130(44.1%) |  |
| COVID-19 infection status |  |  |  | 0.241 |
|  | Not infected | 11(5.9%) | 14(8.3%) |  |
|  | Asymptomatic infection | 3(1.6%) | 1(0.6%) |  |
|  | Mild type | 150(81.1%) | 125(74%) |  |
|  | Common type or more serious | 21(11.4%) | 29(17.2%) |  |
| Working with fever |  |  |  | 0.026* |
|  | Yes | 84(45.4%) | 97(57.4%) |  |
|  | No | 101(54.6%) | 72(42.6%) |  |

Data were N (%).*P<0.05, **P<0.01

Table S3 The condition of depression

| Category | | PHQ-9 <5 | PHQ-9≥5 | P |
| --- | --- | --- | --- | --- |
| Gender |  |  |  | 0.771 |
|  | Male | 24(15.2%) | 33(16.8%) |  |
|  | Female | 134(84.8%) | 163(83.2%) |  |
| Marital status |  |  |  | 0.224 |
|  | Single | 36(22.8%) | 57(29.1%) |  |
|  | Married | 122(77.2%) | 139(70.9%) |  |
| Age |  |  |  | 0.236 |
|  | 18-30 | 50(31.6%) | 63(32.1%) |  |
|  | 31-40 | 54(34.2%) | 81(41.3%) |  |
|  | 41-60 | 54(34.2%) | 52(26.5%) |  |
| Level of Education |  |  |  | 0.191 |
|  | Below undergraduate | 23(14.6%) | 18(9.2%) |  |
|  | Undergraduate | 103(65.2%) | 122(62.2%) |  |
|  | Master | 25(15.8%) | 43(21.9%) |  |
|  | Doctor or above | 7(4.4%) | 13(6.6%) |  |
| Profession |  |  |  | 0.731 |
|  | Medical | 110(69.6%) | 133(67.9%) |  |
|  | Nurse | 48(30.4%) | 63(32.1%) |  |
| Job title |  |  |  | 0.961 |
|  | Junior | 67(42.4%) | 83(42.3%) |  |
|  | Intermediate | 70(44.3%) | 85(43.4%) |  |
|  | Senior | 21(13.3%) | 28(14.3%) |  |
| Department |  |  |  | <0.001** |
|  | Obstetrics | 31(30.7%) | 70(69.3%) |  |
|  | Gynecology | 23(41.1%) | 33(58.9%) |  |
|  | Outpatient | 40(64.5%) | 22(35.5%) |  |
|  | Other | 64(47.4%) | 71(52.6%) |  |
| Wearing protective clothing |  |  |  | 0.252 |
|  | Yes | 22(13.9%) | 37(18.9%) |  |
|  | No | 136(86.1%) | 159(81.1%) |  |
| COVID-19 infection status |  |  |  | 0.405 |
|  | Not infected | 15(9.5%) | 10(5.1%) |  |
|  | Asymptomatic infection | 2(1.3%) | 2(1%) |  |
|  | Mild type | 121(76.6%) | 154(78.6%) |  |
|  | Common type or more serious | 20(12.7%) | 30(15.3%) |  |
| Working with fever |  |  |  | 0.001** |
|  | Yes | 65(35.9%) | 116(64.1%) |  |
|  | No | 93(53.8%) | 80(46.2%) |  |

Data were N (%).**P<0.01

Table S4 The condition of insomnia

| Category | | ISI <8 | ISI≥8 | P |
| --- | --- | --- | --- | --- |
| Gender |  |  |  | 0.759 |
|  | Male | 37(15.6%) | 20(17.1%) |  |
|  | Female | 200(84.4%) | 97(82.9%) |  |
| Marital status |  |  |  | 1.000 |
|  | Single | 62(26.2%) | 31(26.5%) |  |
|  | Married | 175(73.8%) | 86(73.5%) |  |
| Age |  |  |  | 0.857 |
|  | 18-30 | 77(32.5%) | 36(30.8%) |  |
|  | 31-40 | 88(37.1%) | 47(40.2%) |  |
|  | 41-60 | 72(30.4%) | 34(29.1%) |  |
| Level of Education |  |  |  | 0.061 |
|  | Below undergraduate | 35(14.8%) | 6(5.1%) |  |
|  | Undergraduate | 145(61.2%) | 80(68.4%) |  |
|  | Master | 45(19.0%) | 23(19.7%) |  |
|  | Doctor or above | 12(5.0%) | 8(6.8%) |  |
| Profession |  |  |  | 0.716 |
|  | Medical | 161(67.9%) | 82(70.1%) |  |
|  | Nurse | 76(32.1%) | 35(29.9%) |  |
| Job title |  |  |  | 0.210 |
|  | Junior | 108(45.6%) | 42(35.9%) |  |
|  | Intermediate | 97(40.9%) | 58(49.6%) |  |
|  | Senior | 32(13.5%) | 17(14.5%) |  |
| Department |  |  |  | 0.058 |
|  | Obstetrics | 58(24.5%) | 43(36.8%) |  |
|  | Gynecology | 39(16.5%) | 17(14.5%) |  |
|  | Outpatient | 48(20.2%) | 14(12.0%) |  |
|  | Other | 92(38.8%) | 43(36.7%) |  |
| Wearing protective clothing |  |  |  | 1.000 |
|  | Yes | 40(16.9%) | 19(16.2%) |  |
|  | No | 197(83.1%) | 98(83.8%) |  |
| COVID-19 infection status |  |  |  | 0.069 |
|  | Not infected | 21(8.9%) | 4(3.4%) |  |
|  | Asymptomatic infection | 4(1.7%) | 0(0.0%) |  |
|  | Mild type | 183(77.2%) | 92(78.6%) |  |
|  | Common type or more serious | 29(12.2%) | 21(17.9%) |  |
| Working with fever |  |  |  | 0.024* |
|  | Yes | 111(61.3%) | 70(38.7%) |  |
|  | No | 126(72.8%) | 47(27.2%) |  |

Data were N (%).*P<0.05
